# Association between patient ethnicity and prostate cancer diagnosis following a prostate-specific antigen test: a cohort study of 730,000 men in primary care in the UK

**DOI:** 10.1186/s12916-024-03283-5

**Published:** 2024-03-01

**Authors:** Liz Down, Melissa Barlow, Sarah E. R. Bailey, Luke T. A. Mounce, Samuel W. D. Merriel, Jessica Watson, Tanimola Martins

**Affiliations:** 1 Department of Health and Community Sciences, University of Exeter, St Lukes Campus, Heavitree Road, Exeter, EX1 2LU UK; 2 Centre for Primary Care & Health Services Research, University of Manchester, Oxford Road, Manchester, M13 9PL UK; 3 Centre for Academic Primary Care (CAPC), Population Health Sciences, Bristol Medical School, University of Bristol, Canynge Hall, 39 Whatley Road, Bristol, BS8 2PS UK

**Keywords:** Ethnicity, Prostate-specific antigen, Prostate cancer, Blood tests, Health inequities

## Abstract

**Background:**

Black men have higher prostate-specific antigen (PSA) levels and higher prostate cancer incidence and mortality than White men, while Asian men tend to have lower prostate cancer incidence and mortality than White men. Much of the evidence comes from the USA, and information from UK populations is limited.

**Methods:**

This retrospective cohort study used data on patients registered at general practices in England contributing to the Clinical Practice Research Datalink (CPRD) Aurum dataset. Those eligible were men aged 40 and over with a record of ethnicity and a PSA test result recorded between 2010 and 2017 with no prior cancer diagnosis.

The aim was to assess the incidence of prostate cancer following a raised PSA test result in men from different ethnic groups. Additionally, incidence of advanced prostate cancer was investigated. Cancer incidence was estimated from multi-level logistic regression models adjusting for potential confounding factors.

**Results:**

730,515 men with a PSA test were included (88.9% White). Black men and men with mixed ethnicity had higher PSA values, particularly for those aged above 60 years. In the year following a raised PSA result (using age-specific thresholds), Black men had the highest prostate cancer incidence at 24.7% (95% CI 23.3%, 26.2%); Asian men had the lowest at 13.4% (12.2%, 14.7%); incidence for White men was 19.8% (19.4%, 20.2%). The peak incidence of prostate cancer for all groups was in men aged 70–79. Incidence of prostate cancer diagnosed at an advanced stage was similar between Black and White men.

**Conclusions:**

More prostate cancer was diagnosed in Black men with a raised PSA result, but rates of advanced prostate cancer were not higher in this group. In this large primary care-based cohort, the incidence of prostate cancer in men with elevated PSA levels increases with increasing age, even when using age-adjusted thresholds, with Black men significantly more likely to be diagnosed compared to White or Asian men. The incidence of advanced stage prostate cancer at diagnosis was similar for Black and White men with a raised PSA result, but lower for Asian men.

**Supplementary Information:**

The online version contains supplementary material available at 10.1186/s12916-024-03283-5.

## Background

Prostate cancer is common, with an estimated 1.4 million cases and 375,304 deaths reported worldwide in 2020 [1]. Detection of clinically significant prostate cancer at an early stage is important to increase the likelihood that it can be treated effectively [2]. Prior to the COVID-19 pandemic, just over half of all prostate cancers diagnosed in England were at an early stage [3]. There is some debate whether lower urinary tract symptoms (LUTS) are useful in the identification of prostate cancer cases [4]. In the UK, as in many other countries, there is currently no national screening programme for prostate cancer [5] although asymptomatic men aged 50 years and older can request a PSA test from their general practitioner (GP) [6]. Levels of PSA increase with age, and some countries have introduced age-stratified diagnostic ranges for prostate cancer. The National Institute for Health and Care Excellence (NICE) last updated its guidance for PSA thresholds for men with LUTS in England and Wales in 2021, which includes a recommendation for age-specific thresholds ranging from 2.5 to 6.5 ng/ml [7]. For asymptomatic men concerned about possible prostate cancer, the National Health Service (NHS) Prostate Cancer Risk Management Programme (PCRMP) recommended age-specific PSA thresholds between 2008 and 2016 and a fixed threshold of 3 ng/ml between 2016 and 2021 [8].

Several studies have observed that there are differences in average PSA values for men from different ethnic groups [9]. The most consistently observed difference is when comparing Black men with non-Hispanic White men, with the former having higher PSA values on average [9–13]. It is unclear whether the diagnostic performance of PSA for prostate cancer differs for patients from different ethnic groups in the UK. This is important considering there are notable differences in prostate cancer incidence between patients from different ethnic groups.

In the UK and USA, research has shown that Black men have the highest risk of receiving a prostate cancer diagnosis, with Asian men in the UK having the lowest risk, a pattern repeated in the life-time risk of dying from the disease [14–17]. However, a recent UK study showed that Black men may be less likely to be diagnosed at an advanced stage [18], in contrast to evidence from the USA [17, 19]. In the USA, patients’ socio-economic status plays an important role in prostate cancer outcomes, but such evidence has not been replicated in the UK, where access to care is universal.

This study used English primary care-linked patient records to investigate prostate cancer diagnosis following a PSA test and associated risk of advanced disease at diagnosis by ethnic group.

## Methods

### Data sources

The data for this study were provided by the Clinical Practice Research Datalink (CPRD), where primary care records can be linked to a selection of routine healthcare datasets. The CPRD Aurum dataset holds information on patient demographics, ethnicity, suspected cancer symptoms, investigations, and diagnoses [20]. As of May 2022, the CPRD Aurum dataset contains information on 41,200,722 patients in total, and 13,300,067 currently registered patients, representing 19.8% of the UK population [21]. Secondary care data from the Hospital Episode Statistics Admitted Patient Care (HES APC) dataset [22] was used to provide additional information for ethnicity coding. The latest available data on cancer diagnoses and stage from the English National Cancer Registration and Analysis Service (NCRAS) cancer registration dataset [23] were obtained for included patients.

### Study population

The study cohort included men aged 40 years and over who were registered with a GP practice in England contributing data to the CPRD during the study period (2010–2017). Eligible participants had a PSA test carried out during this period, with a valid result recorded. A PSA test result was considered to be valid if the result was greater than 0, and the units recorded were either ng/ml or μg/L. The study period began in 2010 as the completeness of the NCRAS prostate cancer data before this date was low [24]. Individuals with any recorded cancer (except for non-melanoma skin cancer) before the first PSA test date were excluded from the analysis, as were men who died within 1 year of the test date with no prostate cancer diagnosis.

### Outcome variables

Prostate cancer diagnoses within 1 year of PSA test were identified from the NCRAS data, using ICD10 code C61. Prostate cancer stage at diagnosis was defined using the tumour, nodes and metastasis (TNM) classification, with a T stage of 3 or 4 or an M stage of 1 classified as advanced.

### Main exposure variables

Participants’ ethnicity was defined based on the categories used in the UK Census, which aligns with those collected in UK healthcare settings. This comprised White (British, English, Welsh, Scottish, Northern Irish, any other White background), Asian (Indian, Pakistani, Bangladeshi, Chinese, any other Asian background), Black (African, Caribbean, any other Black background), Mixed (White and Black Caribbean, White and Black African, White and Asian, any other Mixed background), and Other (Arab, any other ethnic group). The ethnic groups in HES APC directly map onto the census categories (White, Indian, Pakistani, Bangladeshi, Chinese, Other Asian, Black African, Black Caribbean, Other Black, Mixed, and Other). Information on participants’ ethnicity was obtained from the CPRD using an algorithm described in previous studies [25–27]. For those with missing data in the CPRD, we used HES ethnicity, as detailed in Additional file 1.

Raw PSA values were recoded to binary variables (with categories normal or raised). The PSA tests used in this study were performed at a time when guidance on recommended test thresholds in England was evolving, and a range of thresholds were applied by laboratories: some age-based and some with a single PSA threshold for men of all ages. The primary analyses for this study used the age-specific PSA thresholds, which were introduced by NICE in 2021 [7]: 0–2.5 ng/ml for men aged 40 to 49, 0–3.5 ng/ml for men aged 50 to 59, 0–4.5 ng/ml for men aged 60 to 69, and 0–6.5 ng/ml for men aged 70 to 79. The guidelines suggest the use of clinical judgement for men over 79. The threshold for men aged 70 to 79 (0–6.5 ng/ml) was applied to men over 79 years old, based on advice by clinicians in the study team. Additionally, the analysis was repeated with the fixed threshold of 0–3 ng/ml for all men, as this has been used in the UK during the study period, and in other countries. For each patient, the first PSA test result during the study period was selected as their index test for the study.

### Other covariates

Covariates included in analyses were year of blood test, age in 5-year age-bands, deprivation, alcohol intake, smoking status, and body mass index (BMI). Deprivation was measured using quintiles of the rank of the 2015 Index of Multiple Deprivation score (IMD) [28], which is a composite area-based statistic incorporating information on income, employment, education, health, crime, and housing. A multimorbidity score was calculated for each patient using the Cambridge Multimorbidity Score (CMS) methodology [29]. The CMS score was subsequently categorised into four groups, depending on the morbidity burden—with the lowest category as 0 (no morbidity) and three quantiles of multimorbidity score.

### Analysis

Multi-level logistic regression, clustering patients within GP practices, was used to assess if the predictive value of an abnormal PSA test result varied across ethnic groups. The first analysis examined prostate cancer incidence across ethnic groups within 1 year of a PSA test. A secondary analysis examined the utility of PSA to predict prostate cancer diagnosed at an advanced stage within 1 year of the test date by ethnicity. For both analyses, the marginal distributions of the models were used to obtain estimated cancer incidences across patient groups adjusted for all covariates.

Sample size calculations determined that 1118 patients would be required in each subgroup (ethnicity and 10-year age band) to detect a cancer incidence of 3% with a margin of error of < 1 percentage point. This sample size was achieved for each of the three main ethnic groups (White, Asian, and Black), but not for all age bands in the Mixed and Other ethnic groups. Analyses were conducted using Stata MP version 17.0. Plots were generated using R 4.2.2 “Innocent and Trusting”. Results were reported in accordance with the Strengthening and Reporting of Observational Studies in Epidemiology (STROBE) statement [30] (see Additional file 2).

### Patient and public involvement

The funding application for this research was developed in consultation with an existing Patient and Public Involvement and Engagement (PPIE) group. The main findings were discussed with a public collaborator group specifically recruited for this study, comprising three African men. The group welcomed the study findings and emphasised the need for improved awareness of prostate cancer, especially targeting Black men.

## Results

### Cohort features

**Fig. 1:**
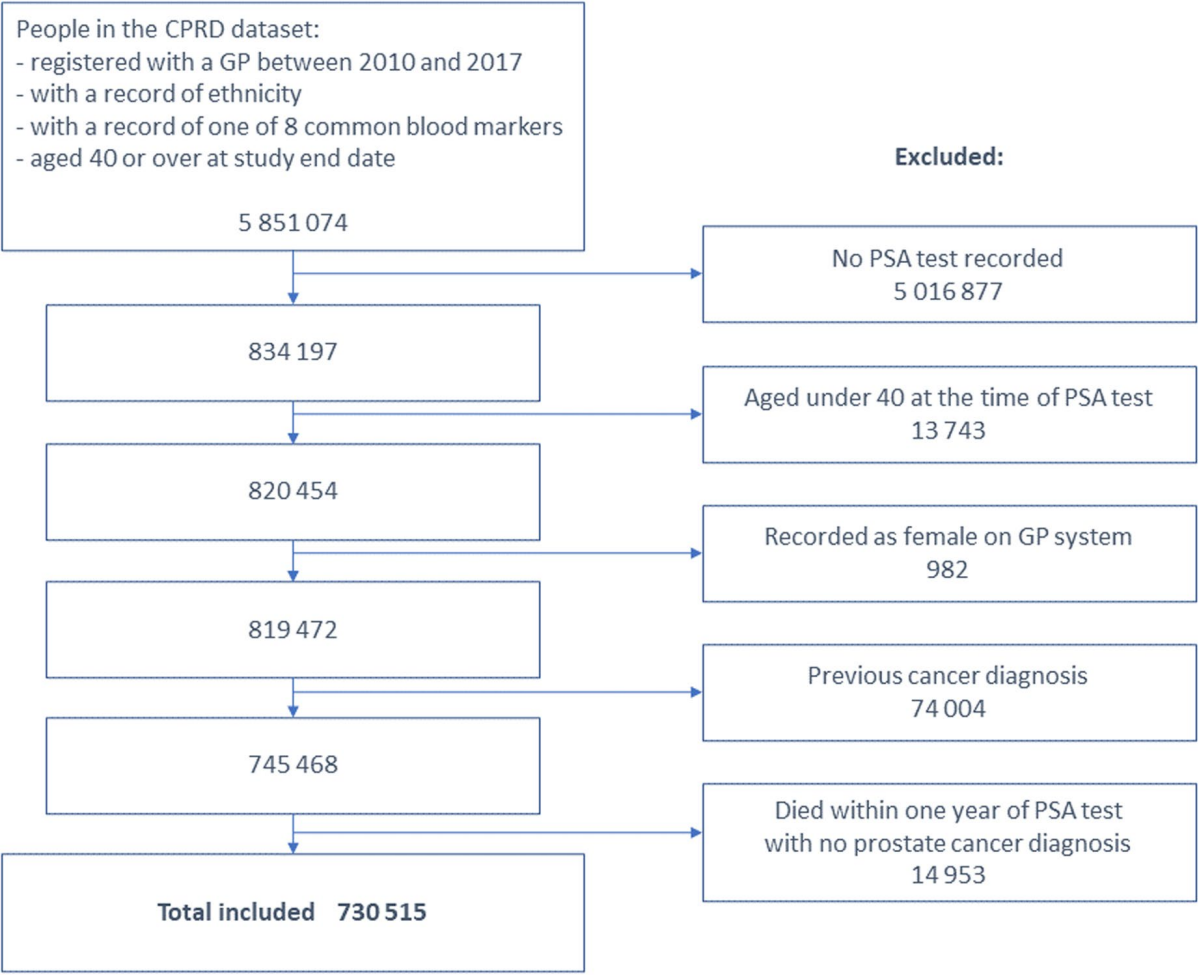
Cohort selection flowchart

The cohort included 730,515 men who had a PSA test (Fig. 1), of which 89% (649 445) were White (Table 1). There was a high degree of heterogeneity between ethnic groups in terms of age distribution and deprivation, with the White group being substantially older and living in less deprived areas than the other groups. Across all age groups, Asian men had the lowest PSA values, with 95th percentile values of 2.2, 4.0, 6.7, 8.8, and 13.3 for the age groups 40–49, 50–59, 60–69, 70–79, and 80 + respectively (Table 2). The highest 95th percentile values were seen in Black men and men of mixed ethnicity at 2.9 (the value for both Black and Mixed groups), 6.0 (Black), 12.9 (Black), 26.9 (Mixed) and 55.4 (Mixed) for the age groups detailed above.

**Table 1:** Population characteristics

	**Number of patients**	**Proportion of cohort**	**Age, median**	**Percent of patients aged 60 or over**	**Percent of patients in most deprived quintile**	**Percent of patients with no multimorbidity**	**Prostate cancer incidence**^**a**^	**Advanced prostate cancer incidence**^**a**^
White	649 445	88.9%	64	64.2%	12.4%	23.3%	24 305 (3.7%)	8 110 (1.2%)
Asian	37 827	5.2%	59	47.7%	22.5%	24.1%	465 (1.2%)	140 (0.4%)
Black	31 053	4.3%	54	33.9%	41.7%	32.5%	1 198 (3.9%)	320 (1.0%)
Mixed	5 736	0.8%	55	37.0%	26.0%	32.0%	155 (2.7%)	36 (0.6%)
Other	6 454	0.9%	56	38.7%	25.5%	36.9%	115 (1.8%)	44 (0.7%)

^a^Unadjusted cancer incidence within 1 year of PSA test. All patients including those with a normal PSA result

**Table 2:** PSA values

Ethnicity	Age group														
	**40–49**			**50–59**			**60–69**			**70–79**			**80 + **		
	95th	Raised (age)	Raised (fixed)	95th	Raised (age)	Raised (fixed)	95th	Raised (age)	Raised (fixed)	95th	Raised (age)	Raised (fixed)	95th	Raised (age)	Raised (fixed)
**White**	2.5	5.0%	3.6%	5.0	9.1%	11.5%	9.0	15.3%	25.7%	14.3	15.5%	37.9%	31.8	25.4%	48.9%
**Asian**	2.2	4.1%	2.9%	4.0	6.4%	8.1%	6.7	10.0%	17.8%	8.8	8.4%	24.6%	13.3	12.9%	32.8%
**Black**	2.9	6.4%	4.8%	6.0	10.4%	13.0%	12.9	19.3%	29.5%	24.0	23.0%	46.6%	42.1	35.6%	58.4%
**Mixed**	2.9	6.5%	4.7%	5.2	9.4%	11.8%	9.6	17.1%	28.8%	26.9	19.7%	43.1%	55.4	23.5%	50.8%
**Other**	2.5	5.3%	3.9%	4.5	8.2%	11.1%	7.6	12.1%	21.6%	15.0	15.5%	34.8%	43.3	19.5%	48.1%

95th = 95th percentile of PSA distribution

Raised (age) = percentage of patients with raised PSA, using age-based thresholds

Raised (fixed) = percentage of patients with raised PSA, using a single fixed threshold

### One-year prostate cancer incidence

**Fig. 2:**
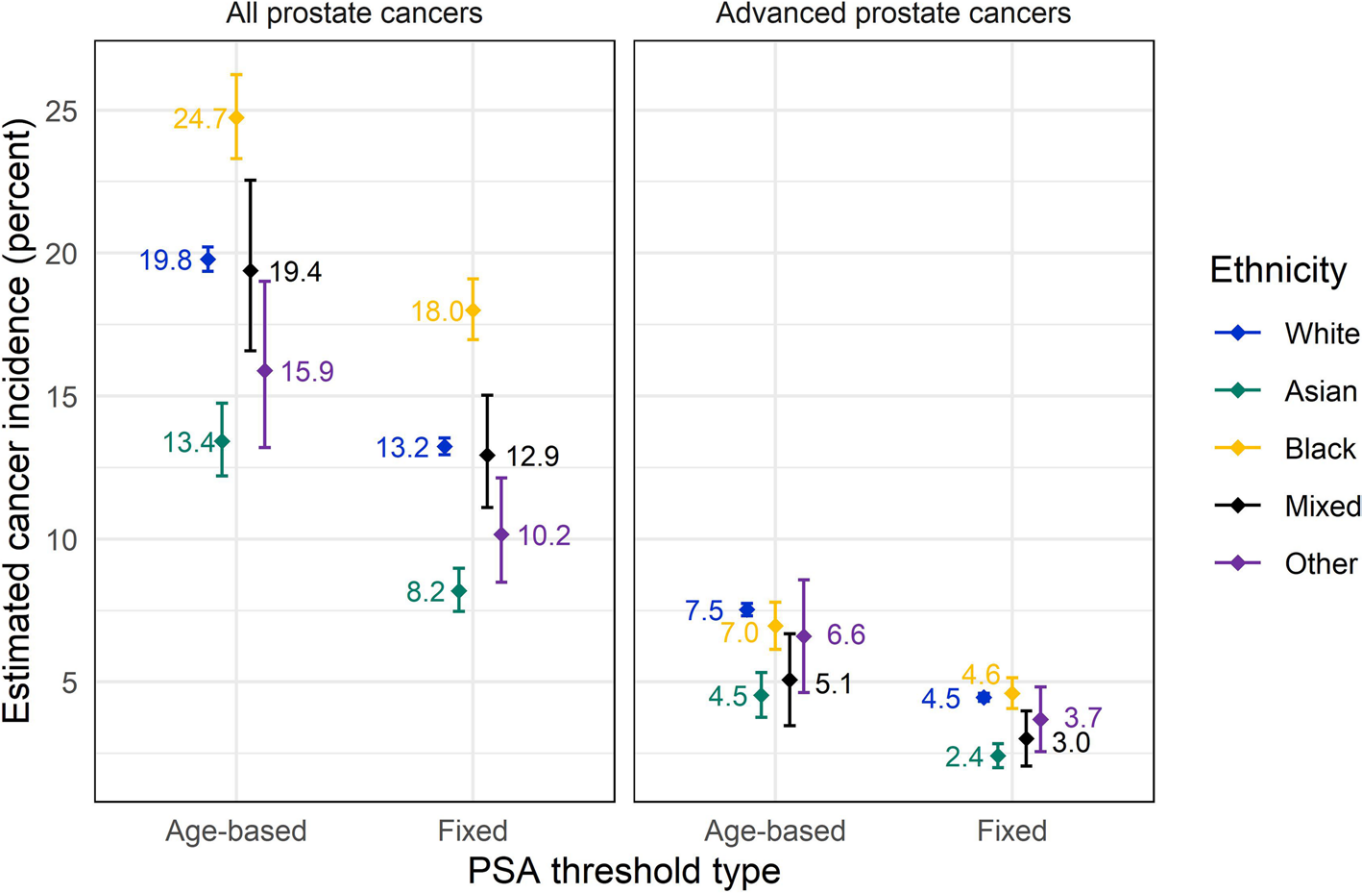
Prostate cancer incidence following a raised PSA test result (adjusted for age and other demographic factors)

Modelling of the risk of prostate cancer diagnosis within 1 year of a PSA test generated prostate cancer incidence estimates adjusted for demographic features. The results of this analysis demonstrated substantial differences between men in the White, Black, and Asian groups (Fig. 2, Additional file 3: Table S4). Using age-based PSA thresholds, 19.8% (95% confidence interval (CI):19.4%, 20.2%) of White men with a raised PSA had a diagnosis of prostate cancer in the following year, compared with 13.4% (95% CI:12.2%, 14.7%) of Asian men and 24.7% (95% CI:23.3%, 26.2%) of Black men. Men in the Mixed and Other ethnic groups had much wider confidence intervals for all outcomes, with an estimated incidence of 19.4% (95% CI: 16.6%, 22.6%) for men in the Mixed group and 15.9% (95% CI:13.2%, 19.0%) for men in the Other group. Analysis using the fixed PSA threshold generated lower cancer incidence estimates for all ethnic groups, but the same relative pattern (Additional file 3: Table S4).

**Fig. 3:**
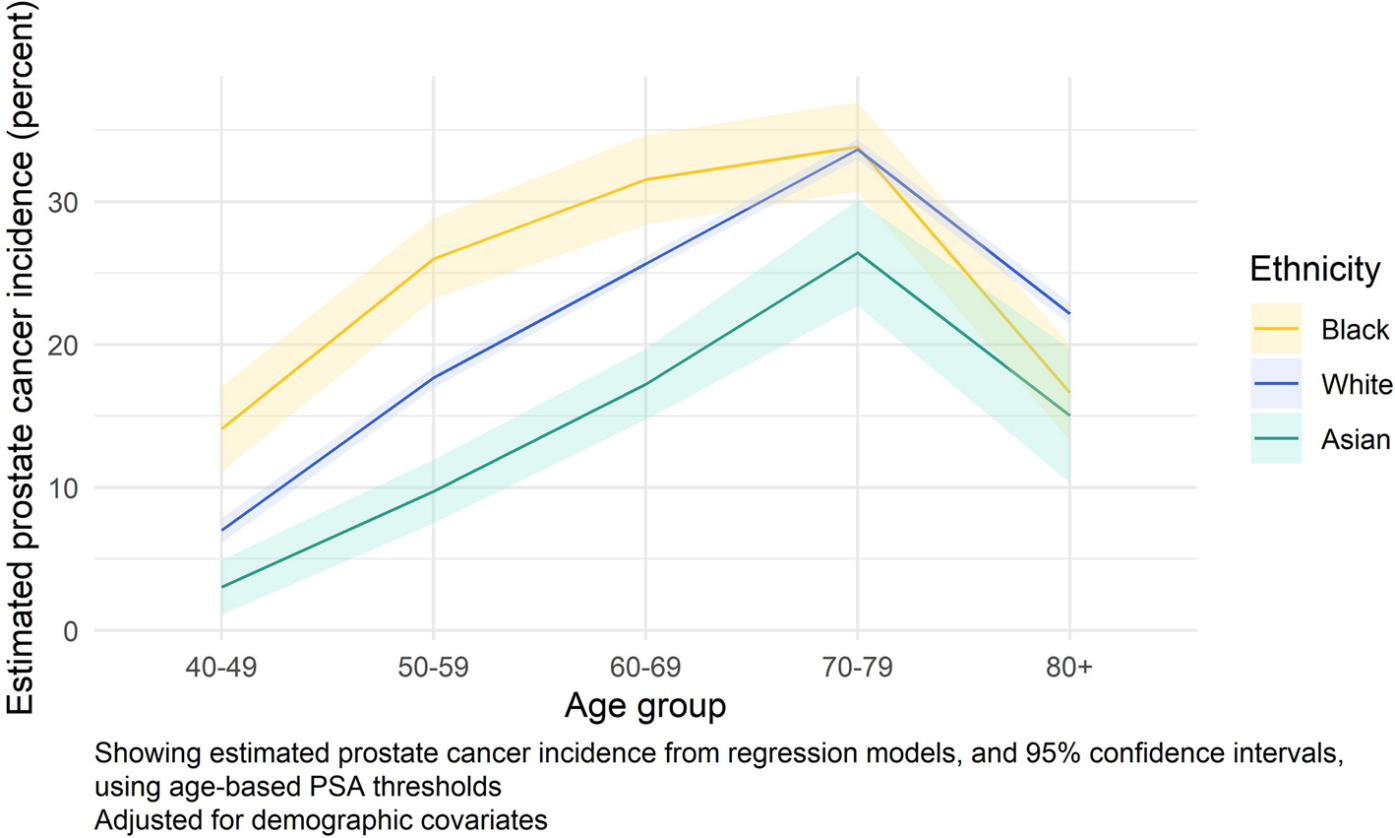
One-year prostate cancer incidence following a raised PSA test result, by age group

One-year prostate cancer incidence stratified by age group for the three largest ethnic groups is illustrated in Fig. 3. In this analysis using age-based PSA thresholds, there was a marked difference in prostate cancer incidence by ethnic group, especially in the youngest age groups (ages 40–69) (Additional file 3: Table S8).

### One-year advanced prostate cancer incidence

Advanced prostate cancer incidence was also investigated using regression modelling (Fig. 2). Asian men had lower incidence of advanced prostate cancer within a year of a raised PSA result compared with White or Black men, both using age-based PSA thresholds and a fixed PSA threshold (Additional file 3: Table S6). Advanced prostate cancer incidence for Asian men with a raised PSA result was 4.5% (95% CI: 3.8%, 5.3%) compared with 7.5% for White men (95% CI: 7.3%, 7.8%) and 7.0% for Black men (95% CI: 6.1%, 7.8%), using age-based thresholds. Incidence estimates for men in the Mixed and Other groups are harder to interpret, as the smaller group size leads to wider confidence intervals.

**Fig. 4:**
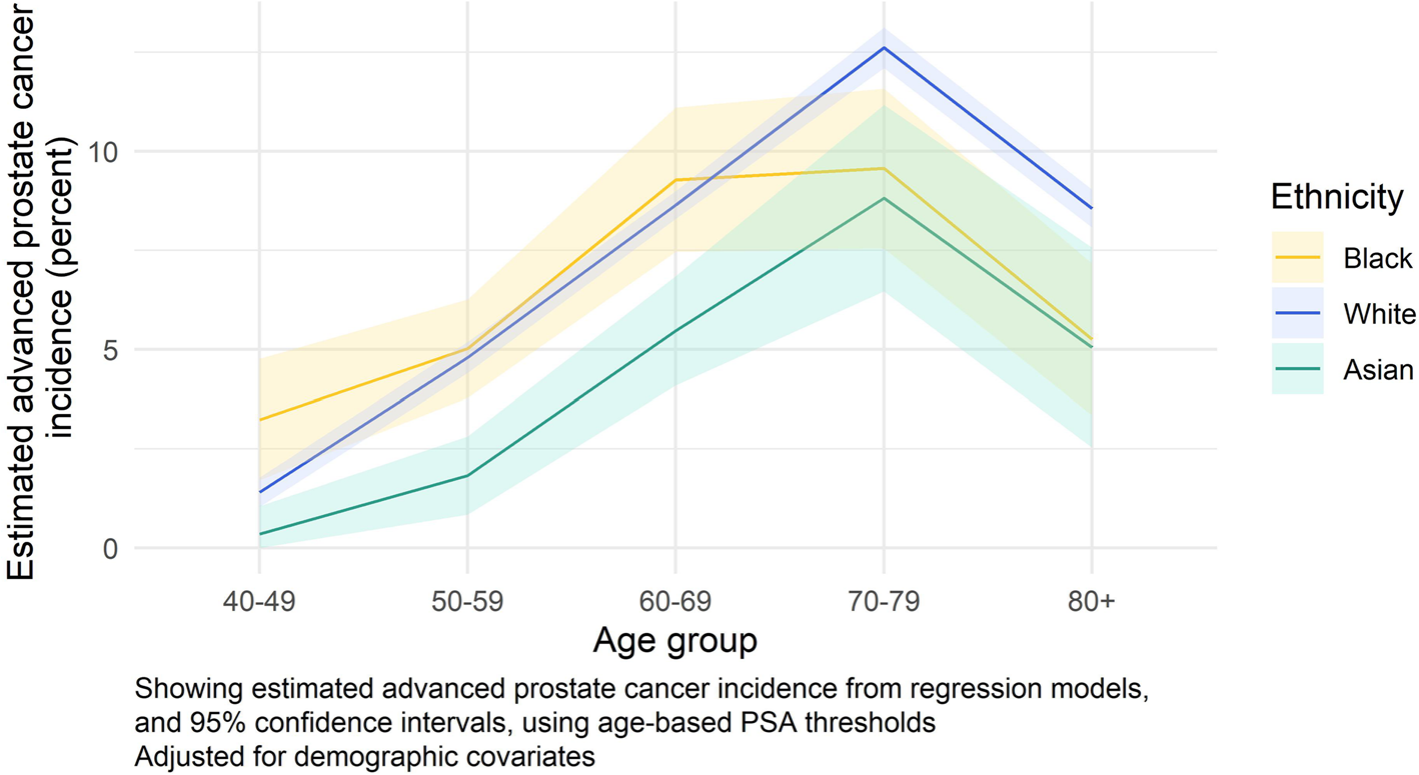
One-year advanced prostate cancer incidence following a raised PSA test result, by age group

When stratified by age group, the 1-year incidence of advanced prostate cancer showed substantial overlap of the confidence intervals across ethnic groups (Fig. 4). White men had the highest incidence of advanced cancer in the oldest age groups (at ages 70–79 and 80 +), while Asian men had the lowest incidence of advanced disease, particularly among those aged 40 to 69 years. In the youngest age group, 40–49 years, Black men had the highest rate of advanced prostate cancer, although the confidence interval at this point does overlap with that of the White group (incidence for White men 1.4% (95% CI: 1.0%, 1.8%); incidence for Black men 3.2% (95% CI: 1.7%, 4.8%)) (Additional file 3: Table S8).

## Discussion

### Main findings

A marked difference in 1-year prostate cancer incidence across ethnic groups was observed. Twenty-five percent of Black men with a raised PSA were diagnosed with prostate cancer within 1 year, compared with 20% of White men and 13% of Asian men. One-year incidence of advanced prostate cancer, however, was similar for Black and White men following a raised PSA result.

### Strengths and weaknesses

This study was based on a large, routinely collected dataset covering approximately 20% of the UK population [21] and a timeframe of 8 years. PSA test results were automatically captured in the primary care data, minimising recording errors. Ethnicity recording in the combined CPRD Aurum and HES dataset was available for more than 90% of patients. The primary care dataset is linked to NCRAS, the definitive record of cancer diagnoses in England.

A limitation of the study was the inability to calculate the false negative rate of PSA, in part because most patients with elevated PSA would be referred for specialist investigation in secondary care while almost all men with normal PSA levels would not. Additionally, it was impossible to fully determine the reasons behind each PSA test in primary care. While there is some degree of opportunistic screening with PSA tests [31, 32], there is no formal screening programme for prostate cancer in the UK. Clinical guidelines recommend PSA testing for men with LUTS and other symptoms associated with prostate cancer. In practice, however, we know that many seemingly asymptomatic men do request PSA screening in UK primary care.

It is probable that some level of bias exists in the level of PSA testing by ethnicity in primary care. For instance, men considered at risk of prostate cancer (aged over 50 years, men with a family history of prostate cancer, and Black men) may be more likely to request or be offered a PSA test and to be offered specialist investigations for a borderline result. This is not something that has been addressed in this study but clearly would benefit from further investigation.

The CPRD data resource is representative of the general population of England in terms of socio-demographic measures and ethnic groups [33, 34]. However, the White and Black groups were slightly over-represented in this cohort, while the Asian, Mixed, and Other ethnic groups were marginally under-represented.

This project used combined ethnic grouping for analyses for simplicity, although it is recognised that this may hide differences between ethnic sub-groups. Furthermore, it was not possible to draw any conclusions from the analyses involving the Mixed and Other ethnic groups due to significant heterogeneity within these groups, relatively younger age and smaller sample sizes, making it difficult to identify any clear patterns.

A limitation of the study is that we did not examine ethnic differences in disease aggressiveness or patient-reported outcomes following a raised PSA. This is an important area for future work. Finally, we did not assess the role of family history of prostate cancer as this factor was not well-recorded in our dataset.

### Comparison with other studies

This is the first UK study to investigate the role of PSA in prostate cancer diagnosis, including advanced-stage disease, by ethnic group.

The observation of higher incidence of prostate cancer in Black men is well known. A recent study in England reported age-standardised incidence rates for Black men at 2.1 times that of White men and age-standardised incidence for Asian men 0.5 times that of White men [15]. In the USA, Siegel et al. [17] showed that Black men (excluding Hispanic men) had age-standardised prostate cancer incidence that was 1.7 times that of White men, while men classified as Asian/Pacific Islander (API) had a prostate cancer incidence 0.7 times that of White men. These figures support those from the current analysis, showing that prostate cancer incidence in Black men was 1.3 times that of White men and Asian men having a prostate cancer incidence that was 0.7 times that of White men.

Previous research on cancer stage at diagnosis gives a more complex picture. For instance, a study using English data [18] found that men classified as Caribbean or African had lower odds of being diagnosed with late-stage disease, compared to White men, while Chinese and Asian men had similar odds compared to White men. A recent study of patients from the USA [19] found that Black men had a higher incidence of regional or distant disease compared to White men and that men in the API group had a lower incidence of regional or distant disease compared to White men but that the proportion of cancers diagnosed at a localised stage is similar.

### Interpretation

Given that most patients who are diagnosed with prostate cancer have slowly developing disease, there is the potential that some men will be diagnosed and treated for prostate cancer, who may not have suffered significant morbidity from the disease during their lifetime. The potential for overdiagnosis and the subsequent psychological and physical impact of diagnosis and treatment is an important consideration [35]. Unlike many other cancer sites, it can be argued that it may not be beneficial for every prostate cancer diagnosis to be treated with curative intent.

Black men are more likely to be diagnosed with prostate cancer than men from other ethnic groups, especially in the younger age groups. However, Black men with raised PSA in this cohort have not experienced higher rates of advanced prostate cancer, which is reassuring. It is possible that the higher levels of PSA seen in Black men are not the result of higher prostate cancer rates but a causal factor, given the nature of prostate cancer as a typically indolent disease. The low rate of advanced cancer seen in Asian men suggests that lower PSA levels in these men are not leading to widespread underdiagnosis of clinically significant disease.

This study observed differences in PSA levels and prostate cancer incidence between ethnic groups but cannot explore reasons for those observed differences. Possible causal factors may include genetic factors, access to healthcare, and other environmental/social causes, covering a wide range of factors such as diet, exercise, and the experience of racism [36]. Genetic differences in prostate cancer susceptibility have been identified [37], but it is unlikely this completely explains the observed effects. UK evidence suggests that men from different ethnic groups are equally likely to seek medical help for LUTS [38]. However, Asian men are less likely to be offered a PSA test [38], and Black men may be reluctant to accept prostate investigations when the perceived risk of prostate cancer is low [39]. Previous research has shown a mixed picture in terms of severity of prostate disease in Black men compared with White men. This may reflect the variety of settings in which data has been collected, different patient populations, and differences in access to healthcare. The near-identical outputs from our models adjusted for available demographic factors and those without suggests that these factors (deprivation, multimorbidity, BMI, smoking and alcohol use) are unlikely to have a substantial role in mediating the effects we identified.

### Unanswered questions and future research

Available ethnicity data does not allow analysis of more detailed ethnic group categories or related elements such as ancestry or country of birth. This information could be valuable to enable a better understanding of possible mechanisms underlying the effects that we have seen. While many studies have examined genetic factors with the potential to impact on PSA testing and prostate cancer diagnosis, the lack of ethnic diversity in genomics datasets is an issue in Western countries.

Further research to investigate the effect of applying different PSA thresholds, either to the population as a whole or to ethnic subgroups, is needed to judge whether adjustments to the current PSA thresholds are appropriate.

## Conclusions

Given the differences in prostate cancer incidence and outcomes between ethnic groups this research gives a valuable insight into the relationship between ethnicity, PSA, and prostate cancer diagnosis in the UK. When designing screening programmes or advice for clinicians on opportunistic screening, it may be necessary to consider the possibility that the identified differences in PSA distribution and prostate cancer incidence in the different ethnic groups may lead to differential under- or over-diagnosis in certain groups. Although early diagnosis of prostate cancer is important to improve outcomes, any testing programme would need to carefully consider this evidence to ensure that new guidelines did not lead to inferior outcomes for people from any ethnic group.

### Supplementary Information

**Additional file 1.** Ethnicity derivation flowchart.

**Additional file 2.** STROBE Statement—completed checklist

**Additional file 3: Table S1.** Demographics. **Table S2.** PSA values. **Table S3.** Unadjusted one-year prostate cancer incidence. **Table S4.** Model output—estimated one-year prostate cancer incidence for men with a raised PSA result. **Table S5.** Model output—estimated one-year prostate cancer incidence for men with a raised PSA result—stratified by age group. **Table S6.** Model output for advanced prostate cancer—estimated one-year advanced prostate cancer incidence for men with a raised PSA result. **Table S7.** Model output for advanced prostate cancer—estimated one-year advanced prostate cancer incidence for men with a raised PSA result—stratified by age group. **Table S8.** Statistics presented in Figs. 3 and 4.

## Data Availability

The data was provided to us under licence from CPRD; therefore, we are unable to share this dataset.

## Abbreviations

API: Asian/Pacific Islander
BMI: Body mass index
CI: Confidence interval
CPRD: Clinical Practice Research Datalink
GP: General practitioner
HES: Hospital Episode Statistics
HES APC: Hospital Episode Statistics Admitted Patient Care
LUTS: Lower urinary tract symptoms
NCRAS: National Cancer Registration and Analysis Service
NHS: National Health Service
NICE: National Institute for Health and Care Excellence
PCRMP: Prostate Cancer Risk Management Programme
PPIE: Patient and public involvement and engagement
PSA: Prostate-specific antigen
TNM: Tumour, nodes, metastases
