# Supplementary figures and images for: Association between patient ethnicity and prostate cancer diagnosis following a prostate-specific antigen test: a cohort study of 730,000 men in primary care in the UK
Source: BMC Med. 2024 Mar 1;22:82. doi: 10.1186/s12916-024-03283-5 (PMC10905783; doi:10.1186/s12916-024-03283-5)

Ethnicity derivation


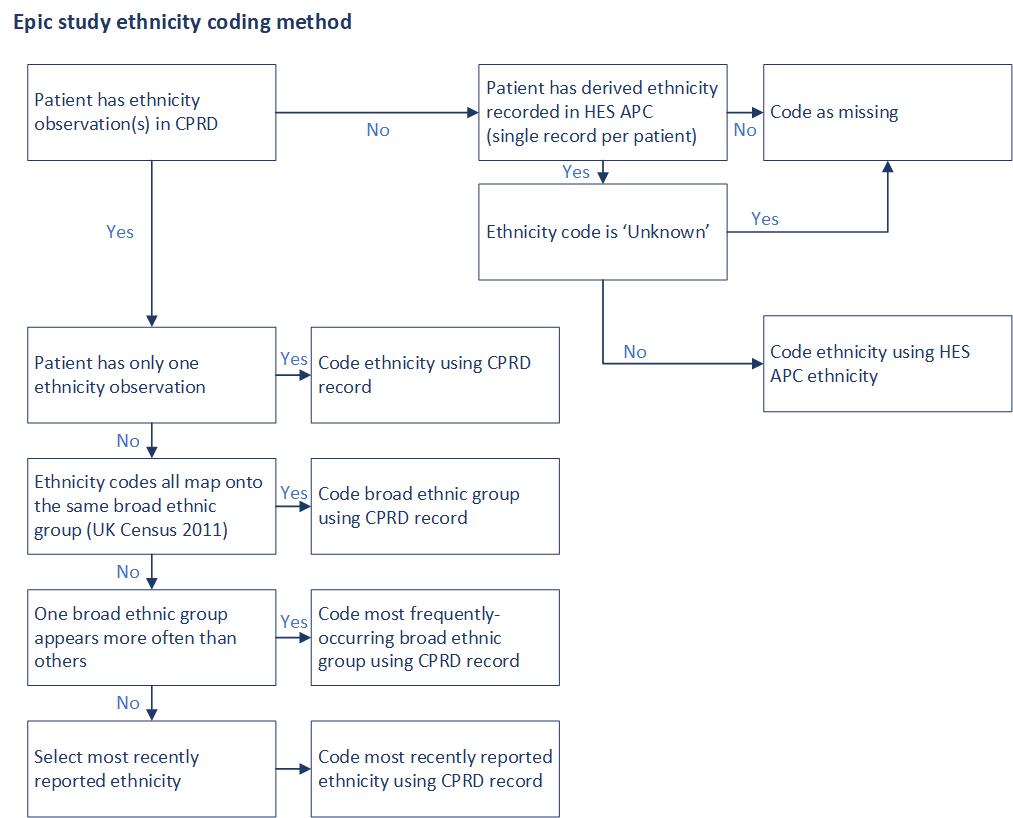

Supplement: Supplementary file 1 — Additional file 1. Ethnicity derivation flowchart. [file 12916_2024_3283_MOESM1_ESM.docx]
