# Supplementary material for: Association between patient ethnicity and prostate cancer diagnosis following a prostate-specific antigen test: a cohort study of 730,000 men in primary care in the UK
Source: BMC Med. 2024 Mar 1;22:82. doi: 10.1186/s12916-024-03283-5 (PMC10905783; doi:10.1186/s12916-024-03283-5)
Supplement: Supplementary file 3 — Additional file 3: Table S1. Demographics. Table S2. PSA values. Table S3. Unadjusted one-year prostate cancer incidence. Table S4. Model output—estimated one-year prostate cancer incidence for men with a raised PSA result. Table S5. Model output—estimated one-year prostate cancer incidence for men with a raised PSA result—stratified by age group. Table S6. Model output for advanced prostate cancer—estimated one-year advanced prostate cancer incidence for men with a raised PSA result. Table S7. Model output for advanced prostate cancer—estimated one-year advanced prostate cancer incidence for men with a raised PSA result—stratified by age group. Table S8. Statistics presented in Figs. 3 and 4. [file 12916_2024_3283_MOESM3_ESM.docx]

**Supplementary Tables**

**Table S1. Demographics**

|  |  | **White** | **Asian** | **Black** | **Other** | **Mixed** |
| --- | --- | --- | --- | --- | --- | --- |
| **Age group** | *40-49* | 11.6% (75590) | 20.5% (7770) | 30.5% (9480) | 27.7% (1787) | 28.8% (1652) |
|  | *50-59* | 24.2% (157118) | 31.7% (12006) | 35.5% (11039) | 33.6% (2171) | 34.2% (1960) |
|  | *60-69* | 31.8% (206695) | 25.4% (9606) | 15.9% (4941) | 23.2% (1500) | 19.3% (1106) |
|  | *70-79* | 21.9% (142038) | 17.2% (6516) | 13.7% (4244) | 11.0% (709) | 13.2% (758) |
|  | *80+* | 10.5% (68004) | 5.1% (1929) | 4.3% (1349) | 4.4% (287) | 4.5% (260) |
|  | *Least deprived 1* | 27.1% (176043) | 14.1% (5315) | 4.0% (1238) | 15.3% (989) | 16.0% (915) |
| **Deprivation** | *2* | 24.1% (156809) | 16.2% (6121) | 6.3% (1953) | 14.4% (932) | 13.6% (778) |
| **quintile** | *3* | 20.4% (132432) | 20.7% (7849) | 15.0% (4668) | 18.4% (1189) | 18.2% (1043) |
|  | *4* | 16.0% (103826) | 26.6% (10046) | 33.0% (10242) | 26.3% (1698) | 26.3% (1507) |
|  | *Most deprived 5* | 12.4% (80335) | 22.5% (8496) | 41.7% (12952) | 25.5% (1646) | 26.0% (1493) |
| **Cambridge**  **Multimorbidity**  **Score (0 score**  **and tertiles)** | *No multimorbidity 0* | 23.3% (151637) | 24.1% (9120) | 32.5% (10098) | 36.9% (2382) | 32.0% (1833) |
|  | *1* | 28.0% (181962) | 23.5% (8897) | 26.9% (8368) | 27.5% (1775) | 28.0% (1607) |
|  | *2* | 24.1% (156816) | 25.5% (9642) | 25.1% (7785) | 20.5% (1321) | 24.4% (1399) |
|  | *Most multimorbidity 3* | 24.5% (159030) | 26.9% (10168) | 15.5% (4802) | 15.1% (976) | 15.6% (897) |
| **BMI** | *Underweight* | 0.3% (1634) | 0.5% (184) | 0.3% (88) | 0.3% (19) | 0.3% (15) |
|  | *Normal* | 11.3% (73597) | 19.9% (7543) | 13.0% (4042) | 10.6% (683) | 13.1% (752) |
|  | *Overweight* | 20.4% (132344) | 26.0% (9848) | 24.2% (7522) | 21.5% (1388) | 22.6% (1298) |
|  | *Obese* | 14.8% (96305) | 11.3% (4269) | 16.5% (5124) | 13.3% (861) | 13.0% (748) |
|  | *Not measured* | 53.2% (345565) | 42.3% (15983) | 46.0% (14277) | 54.3% (3503) | 51.0% (2923) |
| **Smoking** | *Never* | 34.5% (224201) | 52.7% (19945) | 48.6% (15091) | 41.5% (2678) | 42.8% (2457) |
|  | *Ever* | 65.5% (425244) | 47.3% (17882) | 51.4% (15962) | 58.5% (3776) | 57.2% (3279) |
| **Alcohol** | *Alcohol problem* | 10.7% (69233) | 15.4% (5829) | 16.8% (5207) | 15.8% (1018) | 16.5% (945) |
|  | *No alcohol problem recorded* | 89.3% (580212) | 84.6% (31998) | 83.2% (25846) | 84.2% (5436) | 83.5% (4791) |

**Table S2. PSA values**

| **Men with no prostate cancer diagnosis within a year of test** | | | | | | | | | | | | | | | |
| --- | --- | --- | --- | --- | --- | --- | --- | --- | --- | --- | --- | --- | --- | --- | --- |
| **Ethnicity** | **Age group** | | | | | | | | | | | | | | |
|  | **40-49** | | | **50-59** | | | **60-69** | | | **70-79** | | | **80+** | | |
|  | Med | Raised  (age) | Raised  (fixed) | Med | Raised  (age) | Raised  (fixed) | Med | Raised  (age) | Raised  (fixed) | Med | Raised  (age) | Raised  (fixed) | Med | Raised  (age) | Raised  (fixed) |
| **White** | 0.7 | 4.6% | 3.3% | 0.9 | 7.6% | 10.0% | 1.4 | 11.9% | 22.6% | 1.9 | 11.0% | 34.1% | 2.6 | 21.1% | 45.8% |
| **Asian** | 0.7 | 3.9% | 2.8% | 0.8 | 5.8% | 7.6% | 1.1 | 8.5% | 16.3% | 1.4 | 6.4% | 22.7% | 1.7 | 11.1% | 31.3% |
| **Black** | 0.8 | 5.5% | 4.0% | 0.9 | 7.9% | 10.5% | 1.5 | 14.0% | 24.7% | 2.4 | 16.9% | 41.5% | 3.5 | 31.3% | 55.5% |
| **Other** | 0.7 | 5.0% | 3.6% | 0.9 | 7.4% | 10.3% | 1.4 | 9.7% | 19.4% | 1.8 | 11.6% | 31.6% | 2.5 | 15.5% | 45.4% |
| **Mixed** | 0.8 | 6.1% | 4.3% | 0.9 | 7.9% | 10.3% | 1.5 | 13.8% | 26.0% | 2.2 | 13.4% | 38.5% | 2.8 | 19.9% | 48.4% |
| **Men with a localised prostate cancer diagnosis within a year of test** | | | | | | | | | | | | | | | |
| **Ethnicity** | **40-49** | | | **50-59** | | | **60-69** | | | **70-79** | | | **80+** | | |
|  | Med | Raised  (age) | Raised  (fixed) | Med | Raised  (age) | Raised  (fixed) | Med | Raised  (age) | Raised  (fixed) | Med | Raised  (age) | Raised  (fixed) | Med | Raised  (age) | Raised  (fixed) |
| **White** | 4.4 | 90.4% | 82.8% | 6.0 | 91.2% | 95.1% | 7.8 | 91.0% | 97.0% | 10.6 | 82.2% | 96.7% | 19.6 | 91.3% | 96.0% |
| **Asian** | 5.3 | 100.0% | 100.0% | 7.1 | 94.1% | 94.1% | 7.9 | 89.1% | 96.9% | 10.0 | 79.1% | 95.3% | 56.1 | 100.0% | 100.0% |
| **Black** | 6.4 | 96.1% | 90.2% | 6.7 | 96.9% | 99.4% | 9.5 | 95.0% | 99.2% | 11.7 | 86.0% | 98.6% | 19.1 | 95.2% | 100.0% |
| **Other** | 6.0 | 100.0% | 100.0% | 4.3 | 77.8% | 100.0% | 7.7 | 95.0% | 100.0% | 13.7 | 90.0% | 100.0% | - | - | - |
| **Mixed** | 6.5 | 100.0% | 100.0% | 5.4 | 95.0% | 100.0% | 9.3 | 100.0% | 100.0% | 14.7 | 91.3% | 95.7% | 33.9 | 100.0% | 100.0% |
| **Men with an advanced prostate cancer diagnosis within a year of test** | | | | | | | | | | | | | | | |
| **Ethnicity** | **40-49** | | | **50-59** | | | **60-69** | | | **70-79** | | | **80+** | | |
|  | Med | Raised  (age) | Raised  (fixed) | Med | Raised  (age) | Raised  (fixed) | Med | Raised  (age) | Raised  (fixed) | Med | Raised  (age) | Raised  (fixed) | Med | Raised  (age) | Raised  (fixed) |
| **White** | 15.0 | 95.0% | 91.7% | 18.2 | 97.0% | 98.4% | 21.5 | 96.1% | 98.4% | 30.0 | 93.3% | 98.8% | 79.4 | 97.0% | 98.6% |
| **Asian** | 48.7 | 100.0% | 100.0% | 17.3 | 81.3% | 81.3% | 18.1 | 98.1% | 100.0% | 24.9 | 85.2% | 98.1% | 40.0 | 93.3% | 100.0% |
| **Black** | 35.0 | 100.0% | 100.0% | 27.6 | 98.6% | 100.0% | 33.1 | 98.0% | 100.0% | 35.2 | 90.0% | 97.0% | 56.5 | 100.0% | 100.0% |
| **Other** | 102.3 | 100.0% | 100.0% | 39.8 | 100.0% | 100.0% | 48.1 | 100.0% | 100.0% | 22.4 | 93.3% | 100.0% | 114.0 | 88.9% | 88.9% |
| **Mixed** | 64.6 | 100.0% | 100.0% | 28.0 | 100.0% | 100.0% | 17.3 | 100.0% | 100.0% | 31.0 | 100.0% | 100.0% | 308.2 | 100.0% | 100.0% |

Med = median PSA value;

Raised (age) = Percentage of patients with raised PSA, using age-based thresholds;

Raised (fixed) = Percentage of patients with raised PSA, using a single fixed threshold

**Table S3. Unadjusted one-year prostate cancer incidence**

| **Ethnicity** | **Age group** | | | | | | | | | | | | | | |
| --- | --- | --- | --- | --- | --- | --- | --- | --- | --- | --- | --- | --- | --- | --- | --- |
|  | **40-49** | | | **50-59** | | | **60-69** | | | **70-79** | | | **80+** | | |
|  | **n** | **Cancer** | **Advanced** | **N** | **Cancer** | **Advanced** | **n** | **Cancer** | **Advanced** | **N** | **Cancer** | **Advanced** | **n** | **Cancer** | **Advanced** |
| **White** | 75 590 | 4.0 | 0.8 | 157 118 | 17.4 | 4.4 | 206 695 | 42.1 | 13.6 | 142 038 | 60 | 21.3 | 68 004 | 59.5 | 22.2 |
| **Asian** | 7 770 | 1.2 | 0.1 | 12 006 | 6.5 | 1.3 | 9 606 | 18.1 | 5.6 | 6 516 | 24.9 | 8.3 | 1 929 | 21.8 | 7.8 |
| **Black** | 9 480 | 9.7 | 2.2 | 11 039 | 28.6 | 6.3 | 4 941 | 64.6 | 20 | 4 244 | 90 | 23.6 | 1 349 | 66 | 22.2 |
| **Other** | 1 787 | 2.2 | 0.6 | 2 171 | 9.2 | 1.8 | 1 500 | 27.3 | 10 | 709 | 48 | 21.2 | 287 | 55.7 | 31.4 |
| **Mixed** | 1 652 | 3.6 | 0.6 | 1 960 | 16.8 | 2.6 | 1 106 | 38.9 | 12.7 | 758 | 77.8 | 14.5 | 260 | 53.8 | 19.2 |

n = number of patients in subgroup;

cancer = prostate cancer incidence within one year of PSA test / 1000 men with a PSA test;

advanced = advanced prostate cancer incidence within one year of PSA test / 1000 men with a PSA test

**Table S4. Model output – estimated one-year prostate cancer incidence for men with a raised PSA result**

|  | **Age-based PSA thresholds** | | **Fixed 3ng/ml PSA threshold** | |
| --- | --- | --- | --- | --- |
| **Ethnicity** | **Full model** | **Core variables** | **Full model** | **Core variables** |
| **White** | 19.8%  (19.4%, 20.2%) | 19.8%  (19.4%, 20.3%) | 13.2%  (12.9%, 13.5%) | 13.3%  (13.0%, 13.6%) |
| **Asian** | 13.4%  (12.2%, 14.7%) | 13.1%  (11.9%, 14.4%) | 8.2%  (7.5%, 9.0%) | 8.0%  (7.3%, 8.8%) |
| **Black** | 24.7%  (23.3%, 26.2%) | 24.5%  (23.1%, 25.9%) | 18.0%  (17.0%, 19.1%) | 18.0%  (17.0%, 19.1%) |
| **Other** | 15.9%  (13.2%, 19.0%) | 16.0%  (13.3%, 19.1%) | 10.2%  (8.5%, 12.1%) | 10.2%  (8.6%, 12.2%) |
| **Mixed** | 19.4%  (16.6%, 22.5%) | 19.2%  (16.5%, 22.4%) | 12.9%  (11.1%, 15.0%) | 12.9%  (11.1%, 15.0%) |

Full model - controlling for 5-year age group, deprivation, multimorbidity, BMI, alcohol status, smoking status, PSA test year and GP practice;

Core variables model – controlling for 5-year age group, PSA test year and GP practice.

**Table S5. Model output – estimated one-year prostate cancer incidence for men with a raised PSA result – stratified by age group**

Controlled for 5-year age group, deprivation, multimorbidity, BMI, alcohol status, smoking status, PSA test year and GP practice

| **Analysis using age-based PSA thresholds** | | | | | |
| --- | --- | --- | --- | --- | --- |
| **Ethnicity** | **Age group** | | | | |
|  | **40-49** | **50-59** | **60-69** | **70-79** | **80+** |
| **White** | 7% (6.1%, 7.9%) | 17.7% (17%, 18.4%) | 25.6% (25.1%, 26.2%) | 33.7% (33%, 34.4%) | 22.2% (21.4%, 22.9%) |
| **Asian** | 3% (1.1%, 4.9%) | 9.7% (7.5%, 11.9%) | 17.2% (14.8%, 19.7%) | 26.4% (22.7%, 30.1%) | 15% (10.4%, 19.7%) |
| **Black** | 14.1% (11.2%, 17.1%) | 26% (23.1%, 28.8%) | 31.5% (28.4%, 34.7%) | 33.8% (30.7%, 37%) | 16.6% (13.3%, 20%) |
| **Other** | 4.3% (0.1%, 8.5%) | 9.7% (5.2%, 14.3%) | 21.4% (15.7%, 27.1%) | 27.7% (19.4%, 35.9%) | 23.3% (12.4%, 34.2%) |
| **Mixed** | 5.6% (1.2%, 10%) | 17.2% (11.7%, 22.8%) | 21.9% (16.2%, 27.5%) | 36.3% (28.7%, 43.8%) | 19.7% (9.3%, 30.1%) |
| **Analysis using a fixed PSA threshold** | | | | | |
|  | **Age group** |  |  |  |  |
| **Ethnicity** | **40-49** | **50-59** | **60-69** | **70-79** | **80+** |
| **White** | 9.1% (7.9%, 10.3%) | 14.4% (13.8%, 15%) | 16% (15.7%, 16.4%) | 15.6% (15.2%, 15.9%) | 11.9% (11.4%, 12.3%) |
| **Asian** | 4.2% (1.6%, 6.9%) | 7.7% (5.9%, 9.5%) | 10.1% (8.6%, 11.6%) | 10.1% (8.6%, 11.5%) | 6.3% (4.3%, 8.3%) |
| **Black** | 17.9% (14.2%, 21.7%) | 21.2% (18.9%, 23.6%) | 21.1% (18.9%, 23.4%) | 19.1% (17.1%, 21%) | 10.6% (8.4%, 12.7%) |
| **Other** | 5.8% (0.3%, 11.4%) | 7.9% (4.4%, 11.4%) | 12.2% (8.5%, 15.9%) | 13.1% (9.2%, 17.1%) | 10.3% (5.4%, 15.2%) |
| **White** | 7.7% (1.8%, 13.7%) | 13.9% (9.5%, 18.4%) | 13.5% (9.8%, 17.1%) | 17.7% (13.4%, 21.9%) | 9.5% (4.5%, 14.5%) |

**Table S6. Model output for advanced prostate cancer – estimated one-year advanced prostate cancer incidence for men with a raised PSA result**

|  | **Age-based PSA thresholds** | | **Fixed 3ng/ml PSA threshold** | |
| --- | --- | --- | --- | --- |
| **Ethnicity** | **Full model** | **Core variables** | **Full model** | **Core variables** |
| **White** | 7.5% (7.3%, 7.8%) | 7.5% (7.3%, 7.8%) | 4.5% (4.3%, 4.6%) | 4.5% (4.3%, 4.6%) |
| **Asian** | 4.5% (3.8%, 5.3%) | 4.4% (3.6%, 5.1%) | 2.4% (2.0%, 2.8%) | 2.3% (1.9%, 2.7%) |
| **Black** | 7.0% (6.1%, 7.8%) | 6.9% (6.1%, 7.7%) | 4.6% (4.1%, 5.1%) | 4.6% (4.1%, 5.2%) |
| **Other** | 6.6% (4.6%, 8.6%) | 6.6% (4.7%, 8.6%) | 3.7% (2.6%, 4.8%) | 3.7% (2.6%, 4.9%) |
| **Mixed** | 5.1% (3.5%, 6.7%) | 5.0% (3.4%, 6.6%) | 3.0% (2.1%, 4.0%) | 3.0% (2.0%, 4.0%) |

Full model - controlling for 5-year age group, deprivation, multimorbidity, BMI, alcohol status, smoking status, PSA test year and GP practice;

Core variables model – controlling for 5-year age group, PSA test year and GP practice.

**Table S7. Model output for advanced prostate cancer – estimated one-year advanced prostate cancer incidence for men with a raised PSA result – stratified by age group**

Controlled for 5-year age group, deprivation, multimorbidity, BMI, alcohol status, smoking status, PSA test year and GP practice

| **Analysis using age-based PSA thresholds** | | | | | |
| --- | --- | --- | --- | --- | --- |
| **Ethnicity** | **Age group** | | | | |
|  | **40-49** | **50-59** | **60-69** | **70-79** | **80+** |
| **White** | 1.4% (1%, 1.8%) | 4.8% (4.4%, 5.2%) | 8.6% (8.3%, 9%) | 12.6% (12.1%, 13.1%) | 8.6% (8.1%, 9%) |
| **Asian** | 0.4% (0%, 1.1%) | 1.8% (0.8%, 2.8%) | 5.5% (4.1%, 6.8%) | 8.8% (6.5%, 11.2%) | 5.1% (2.5%, 7.6%) |
| **Black** | 3.2% (1.7%, 4.8%) | 5% (3.8%, 6.3%) | 9.3% (7.5%, 11.1%) | 9.6% (7.5%, 11.6%) | 5.3% (3.3%, 7.2%) |
| **Other** | 1% (0%, 2.9%) | 2.2% (0%, 4.5%) | 7.7% (4.1%, 11.3%) | 11.5% (5.5%, 17.4%) | 11.5% (3.6%, 19.4%) |
| **Mixed** | 0.9% (0%, 2.6%) | 2.5% (0.4%, 4.6%) | 6.9% (3.4%, 10.5%) | 7% (3.1%, 10.8%) | 7.9% (1.3%, 14.4%) |
| **Analysis using a fixed PSA threshold** | | | | | |
|  | **Age group** |  |  |  |  |
| **Ethnicity** | **40-49** | **50-59** | **60-69** | **70-79** | **80+** |
| **White** | 1.9% (1.4%, 2.4%) | 3.8% (3.5%, 4.1%) | 5.3% (5.1%, 5.5%) | 5.6% (5.4%, 5.8%) | 4.5% (4.3%, 4.8%) |
| **Asian** | 0.5% (0%, 1.4%) | 1.4% (0.6%, 2.2%) | 3.1% (2.3%, 3.9%) | 3.4% (2.5%, 4.3%) | 2.1% (1%, 3.2%) |
| **Black** | 4.4% (2.4%, 6.4%) | 4% (3%, 5%) | 6.1% (4.9%, 7.4%) | 5.2% (4.1%, 6.3%) | 3.3% (2.1%, 4.5%) |
| **Other** | 1.3% (0%, 3.9%) | 1.6% (0%, 3.2%) | 4.2% (2.1%, 6.3%) | 5.6% (2.7%, 8.4%) | 5% (1.6%, 8.5%) |
| **Mixed** | 1.3% (0%, 3.7%) | 1.9% (0.3%, 3.4%) | 4.3% (2.1%, 6.6%) | 3.4% (1.5%, 5.3%) | 3.6% (0.6%, 6.6%) |

**Table S8. Statistics presented in figures 3 and 4**

**Model output – estimated one-year prostate cancer incidence for men with a raised PSA result, by age group, using age-based thresholds**

Controlling for 5-year age group, deprivation, multimorbidity, BMI, alcohol status, smoking status, PSA test year and GP practice

| **Figure 3. One-year prostate cancer incidence – estimates from models (95% confidence intervals)** | | | | | |
| --- | --- | --- | --- | --- | --- |
|  | **Age group** | | | | |
| **Ethnicity** | **40-49** | **50-59** | **60-69** | **70-79** | **80+** |
| **White** | 7%  (6.1%, 7.9%) | 17.7%  (17%, 18.4%) | 25.6%  (25.1%, 26.2%) | 33.7%  (33%, 34.4%) | 22.2%  (21.4%, 22.9%) |
| **Asian** | 3%  (1.1%, 4.9%) | 9.7%  (7.5%, 11.9%) | 17.2%  (14.8%, 19.7%) | 26.4%  (22.7%, 30.1%) | 15%  (10.4%, 19.7%) |
| **Black** | 14.1%  (11.2%, 17.1%) | 26%  (23.1%, 28.8%) | 31.5%  (28.4%, 34.7%) | 33.8%  (30.7%, 37%) | 16.6%  (13.3%, 20%) |
|  |  |  |  |  |  |
| **Figure 4. One-year advanced prostate cancer incidence – estimates from models (95% confidence intervals)** | | | | | |
|  | **Age group** | | | | |
| **Ethnicity** | **40-49** | **50-59** | **60-69** | **70-79** | **80+** |
| **White** | 1.4%  (1%, 1.8%) | 4.8%  (4.4%, 5.2%) | 8.6%  (8.3%, 9%) | 12.6%  (12.1%, 13.1%) | 8.6%  (8.1%, 9%) |
| **Asian** | 0.4%  (0%, 1.1%) | 1.8%  (0.8%, 2.8%) | 5.5%  (4.1%, 6.8%) | 8.8%  (6.5%, 11.2%) | 5.1%  (2.5%, 7.6%) |
| **Black** | 3.2%  (1.7%, 4.8%) | 5%  (3.8%, 6.3%) | 9.3%  (7.5%, 11.1%) | 9.6%  (7.5%, 11.6%) | 5.3%  (3.3%, 7.2%) |
